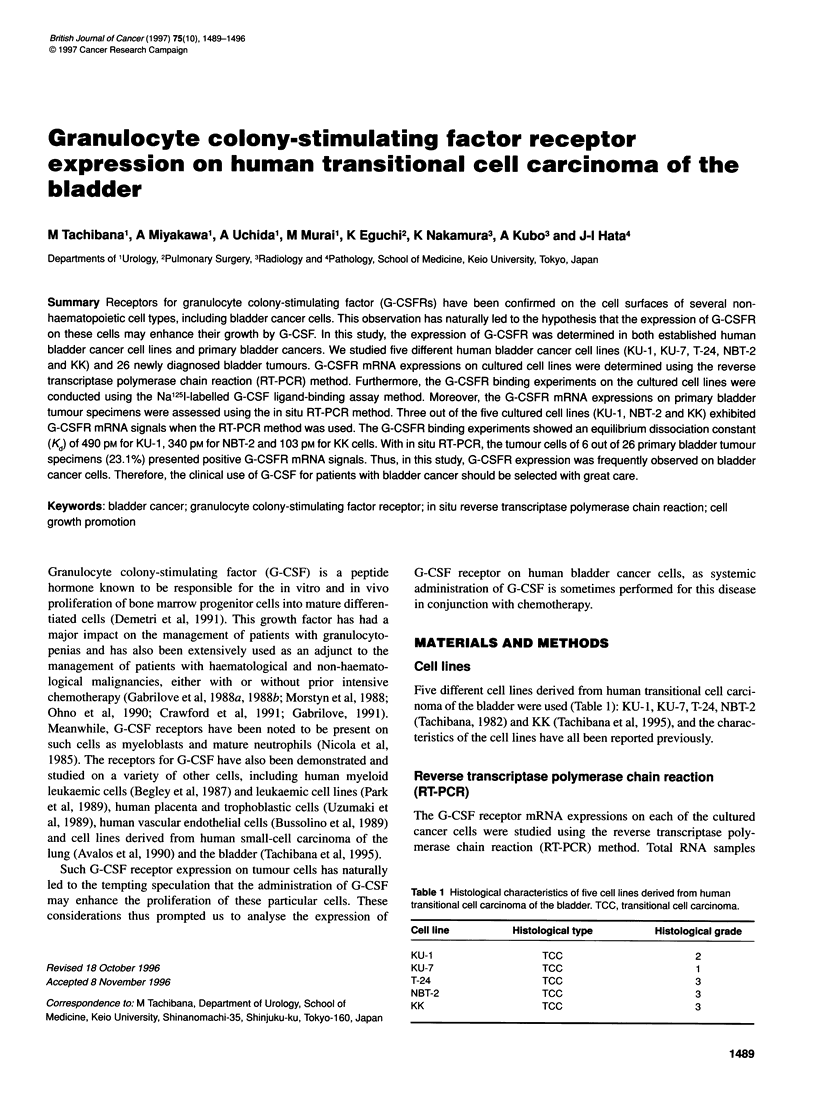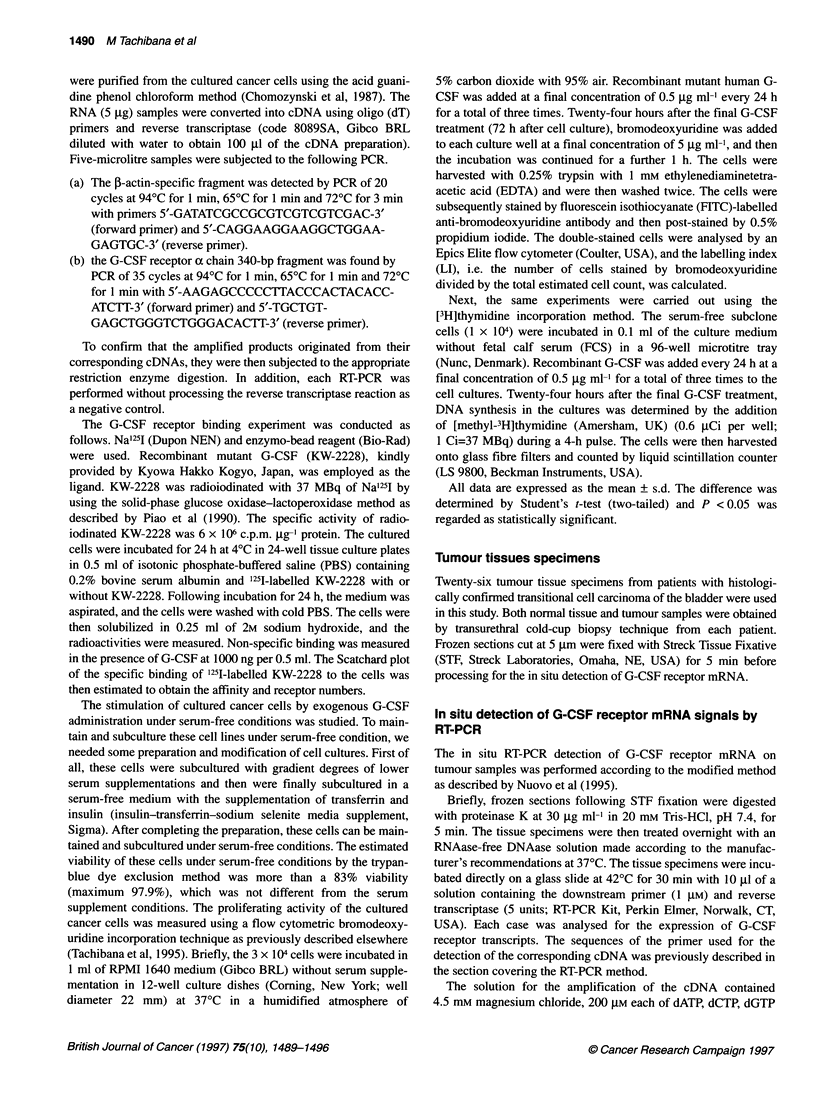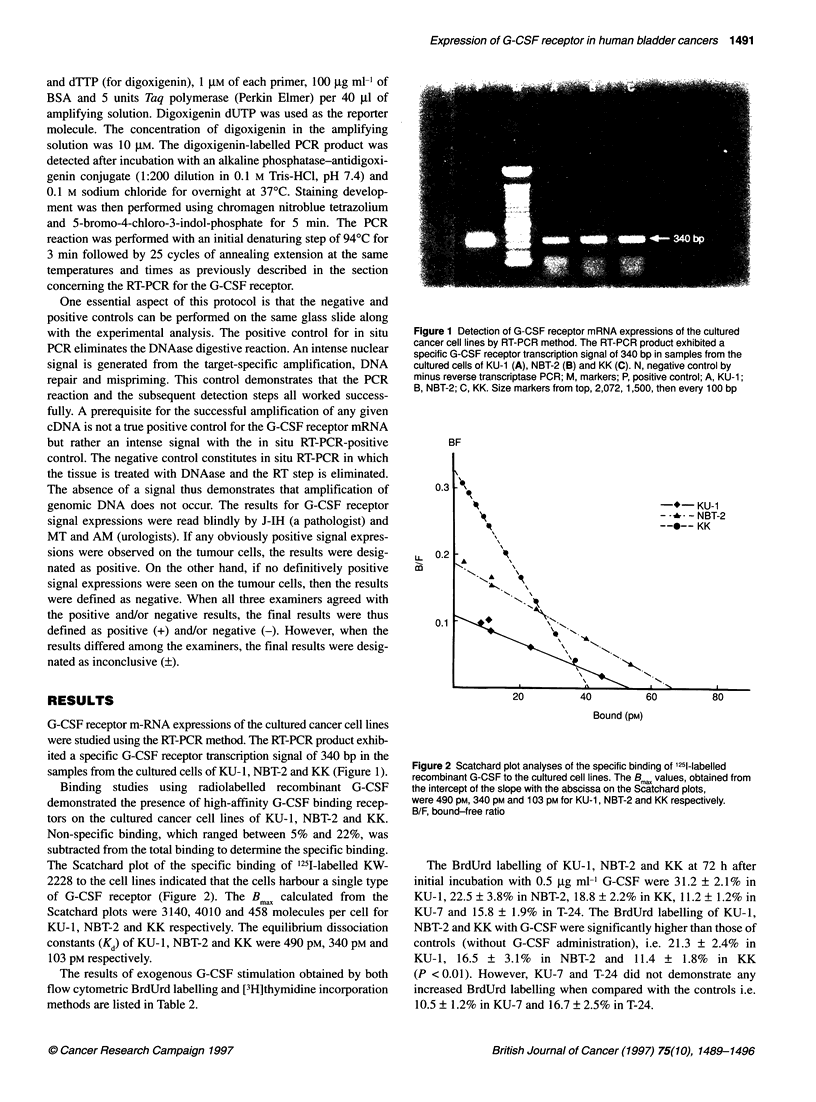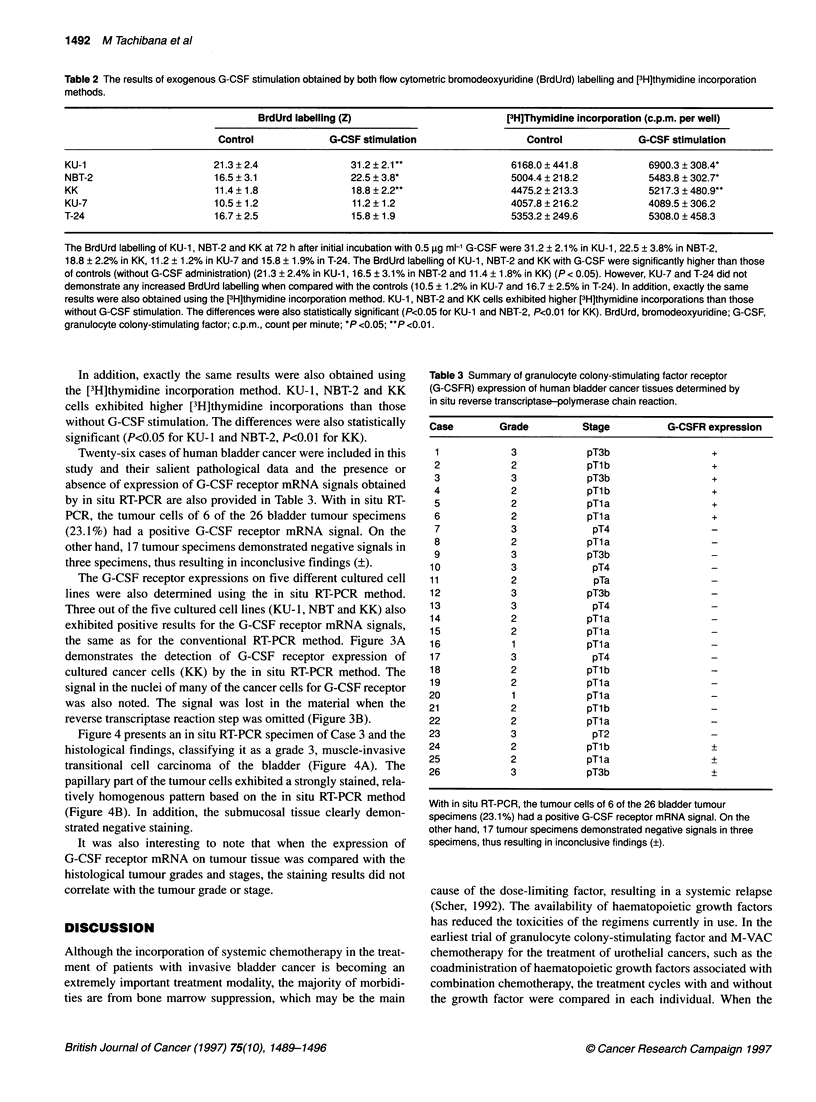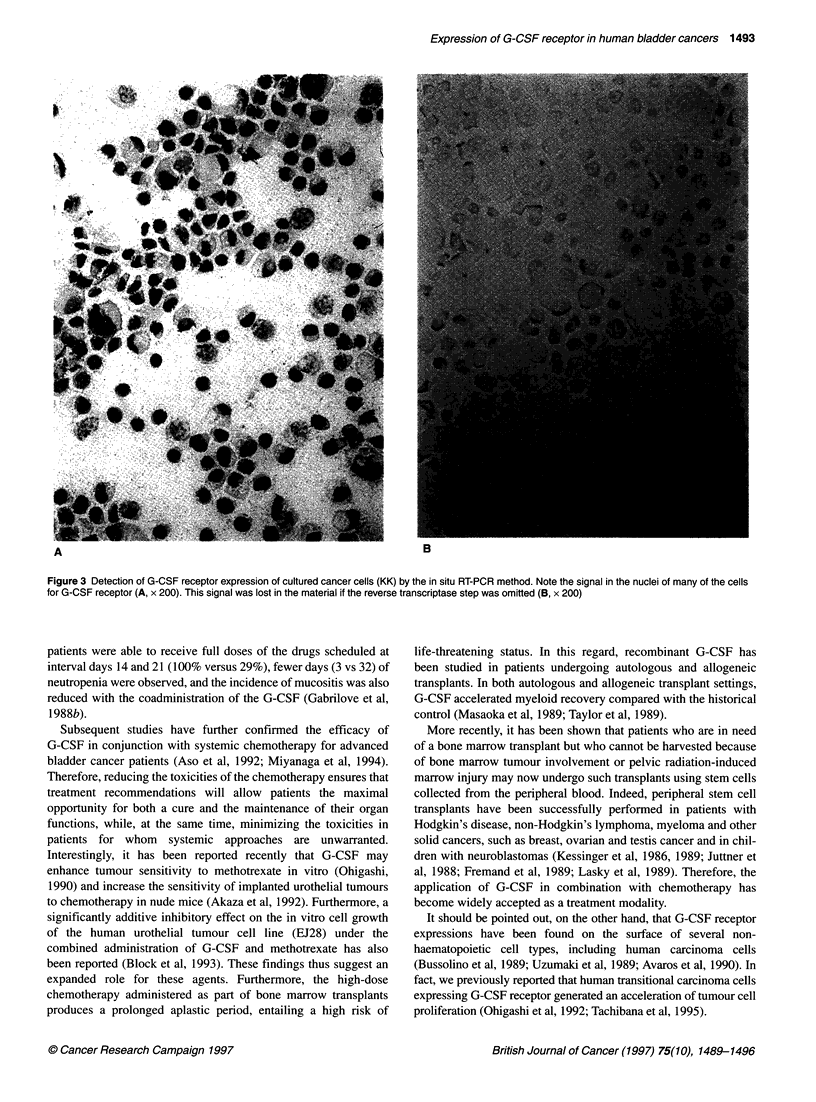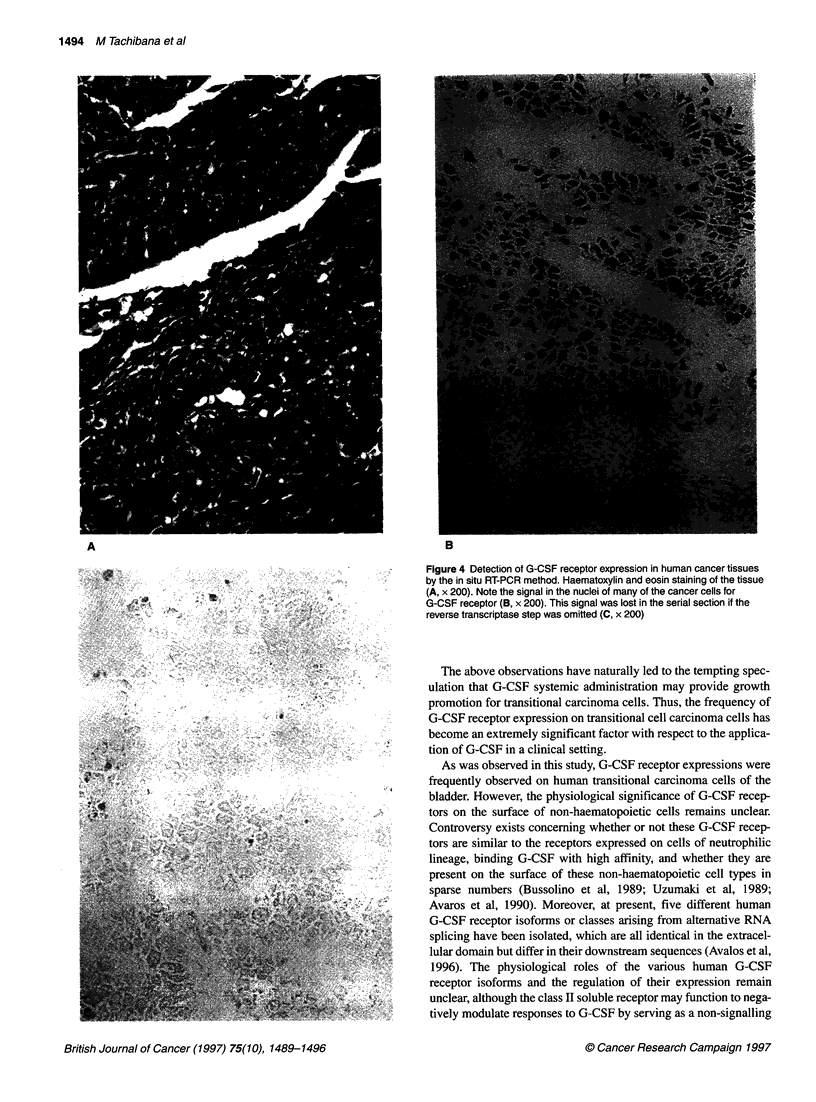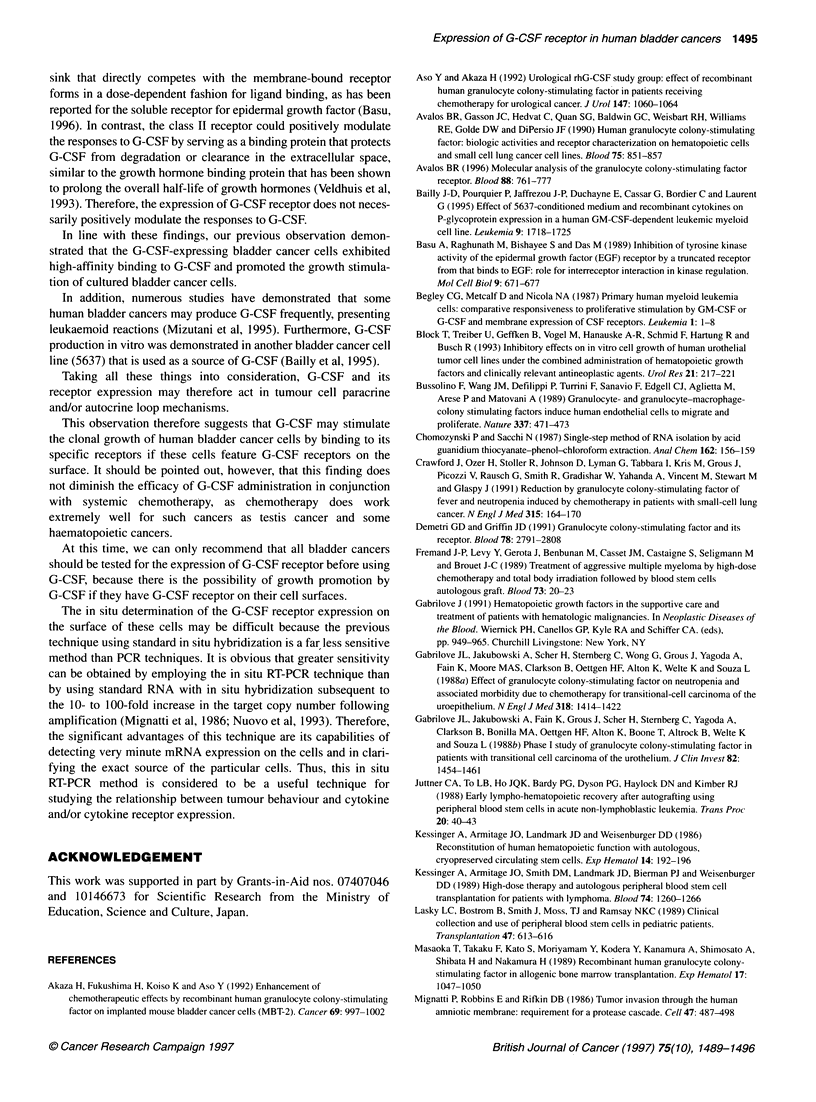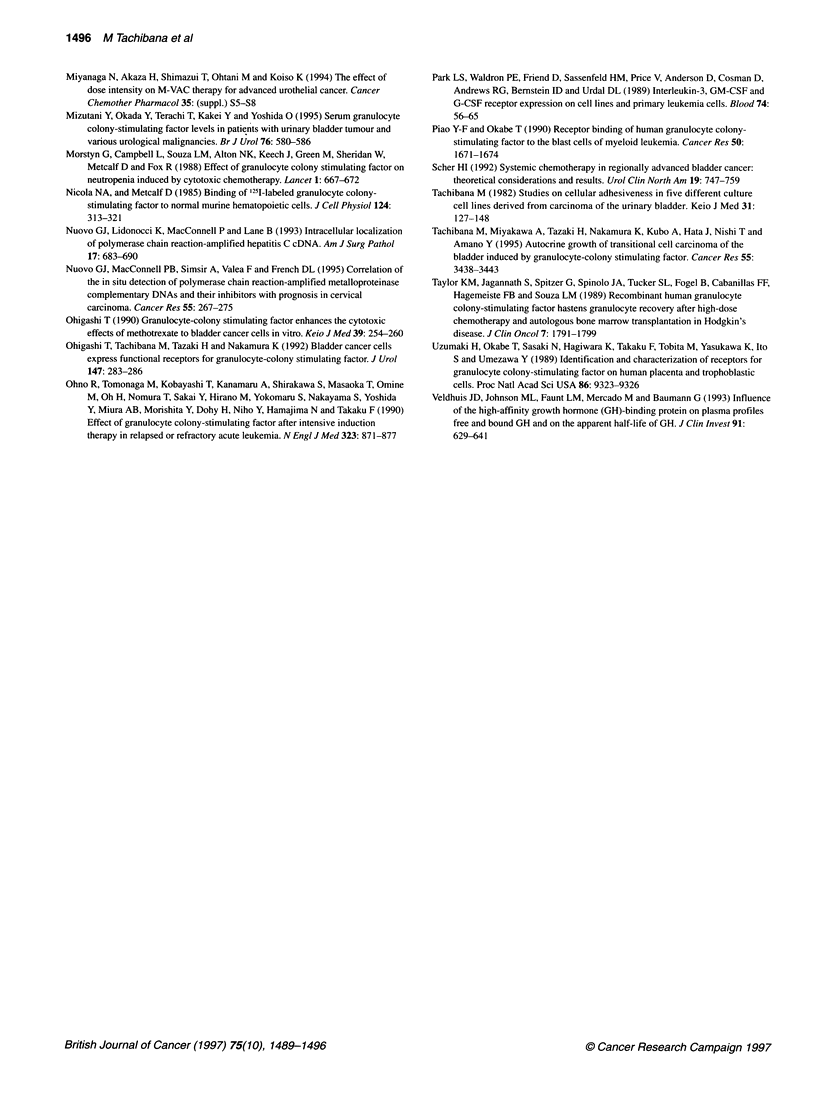# Granulocyte colony-stimulating factor receptor expression on human transitional cell carcinoma of the bladder.

**DOI:** 10.1038/bjc.1997.254

**Published:** 1997

**Authors:** M. Tachibana, A. Miyakawa, A. Uchida, M. Murai, K. Eguchi, K. Nakamura, A. Kubo, J. I. Hata

**Affiliations:** Department of Urology, School of Medicine, Keio University, Tokyo, Japan.

## Abstract

**Images:**


British Journal of Cancer (1997) 75(10), 1489–1496
© 1997 Cancer Research Campaign

### Granulocyte colony-stimulating factor receptor expression on human transitional cell carcinoma of the bladder

**M Tachibana[1], A Miyakawa[1], A Uchida[1], M Murai[1], K Eguchi[2], K Nakamura[3], A Kubo[3] and J-I Hata[4]**

Departments of [1]Urology, [2]Pulmonary Surgery, [3]Radiology and [4]Pathology, School of Medicine, Keio University, Tokyo, Japan

**Summary** Receptors for granulocyte colony-stimulating factor (G-CSFRs) have been confirmed on the cell surfaces of several non-haematopoietic cell types, including bladder cancer cells. This observation has naturally led to the hypothesis that the expression of G-CSFR on these cells may enhance their growth by G-CSF. In this study, the expression of G-CSFR was determined in both established human bladder cancer cell lines and primary bladder cancers. We studied five different human bladder cancer cell lines (KU-1, KU-7, T-24, NBT-2 and KK) and 26 newly diagnosed bladder tumours. G-CSFR mRNA expressions on cultured cell lines were determined using the reverse transcriptase polymerase chain reaction (RT-PCR) method. Furthermore, the G-CSFR binding experiments on the cultured cell lines were conducted using the $Na^{125}I$-labelled G-CSF ligand-binding assay method. Moreover, the G-CSFR mRNA expressions on primary bladder tumour specimens were assessed using the in situ RT-PCR method. Three out of the five cultured cell lines (KU-1, NBT-2 and KK) exhibited G-CSFR mRNA signals when the RT-PCR method was used. The G-CSFR binding experiments showed an equilibrium dissociation constant ($K_d$) of 490 pM for KU-1, 340 pM for NBT-2 and 103 pM for KK cells. With in situ RT-PCR, the tumour cells of 6 out of 26 primary bladder tumour specimens (23.1%) presented positive G-CSFR mRNA signals. Thus, in this study, G-CSFR expression was frequently observed on bladder cancer cells. Therefore, the clinical use of G-CSF for patients with bladder cancer should be selected with great care.

**Keywords**: bladder cancer; granulocyte colony-stimulating factor receptor; in situ reverse transcriptase polymerase chain reaction; cell growth promotion

Granulocyte colony-stimulating factor (G-CSF) is a peptide hormone known to be responsible for the in vitro and in vivo proliferation of bone marrow progenitor cells into mature differentiated cells (Demetri et al, 1991). This growth factor has had a major impact on the management of patients with granulocytopenias and has also been extensively used as an adjunct to the management of patients with haematological and non-haematological malignancies, either with or without prior intensive chemotherapy (Gabrilove et al, 1988*a*, 1988*b*; Morstyn et al, 1988; Ohno et al, 1990; Crawford et al, 1991; Gabrilove, 1991). Meanwhile, G-CSF receptors have been noted to be present on such cells as myeloblasts and mature neutrophils (Nicola et al, 1985). The receptors for G-CSF have also been demonstrated and studied on a variety of other cells, including human myeloid leukaemic cells (Begley et al, 1987) and leukaemic cell lines (Park et al, 1989), human placenta and trophoblastic cells (Uzumaki et al, 1989), human vascular endothelial cells (Bussolino et al, 1989) and cell lines derived from human small-cell carcinoma of the lung (Avalos et al, 1990) and the bladder (Tachibana et al, 1995).

Such G-CSF receptor expression on tumour cells has naturally led to the tempting speculation that the administration of G-CSF may enhance the proliferation of these particular cells. These considerations thus prompted us to analyse the expression of G-CSF receptor on human bladder cancer cells, as systemic administration of G-CSF is sometimes performed for this disease in conjunction with chemotherapy.

*Revised 18 October 1996*
*Accepted 8 November 1996*

*Correspondence to:* M Tachibana, Department of Urology, School of Medicine, Keio University, Shinanomachi-35, Shinjuku-ku, Tokyo-160, Japan

### MATERIALS AND METHODS

### Cell lines

Five different cell lines derived from human transitional cell carcinoma of the bladder were used (Table 1): KU-1, KU-7, T-24, NBT-2 (Tachibana, 1982) and KK (Tachibana et al, 1995), and the characteristics of the cell lines have all been reported previously.

### Reverse transcriptase polymerase chain reaction (RT-PCR)

The G-CSF receptor mRNA expressions on each of the cultured cancer cells were studied using the reverse transcriptase polymerase chain reaction (RT-PCR) method. Total RNA samples

**Table 1** Histological characteristics of five cell lines derived from human transitional cell carcinoma of the bladder. TCC, transitional cell carcinoma.

| Cell line | Histological type | Histological grade |
|---|---|---|
| KU-1 | TCC | 2 |
| KU-7 | TCC | 1 |
| T-24 | TCC | 3 |
| NBT-2 | TCC | 3 |
| KK | TCC | 3 |

were purified from the cultured cancer cells using the acid guanidine phenol chloroform method (Chomozynski et al, 1987). The RNA (5 μg) samples were converted into cDNA using oligo (dT) primers and reverse transcriptase (code 8089SA, Gibco BRL diluted with water to obtain 100 μl of the cDNA preparation). Five-microlitre samples were subjected to the following PCR.

(a) The β-actin-specific fragment was detected by PCR of 20 cycles at 94°C for 1 min, 65°C for 1 min and 72°C for 3 min with primers 5′-GATATCGCCGCGTCGTCGTCGAC-3′ (forward primer) and 5′-CAGGAAGGAAGGCTGGAA-GAGTGC-3′ (reverse primer).
(b) the G-CSF receptor α chain 340-bp fragment was found by PCR of 35 cycles at 94°C for 1 min, 65°C for 1 min and 72°C for 1 min with 5′-AAGAGCCCCCTTACCCACTACACC-ATCTT-3′ (forward primer) and 5′-TGCTGT-GAGCTGGGTCTGGGACACTT-3′ (reverse primer).

To confirm that the amplified products originated from their corresponding cDNAs, they were then subjected to the appropriate restriction enzyme digestion. In addition, each RT-PCR was performed without processing the reverse transcriptase reaction as a negative control.

The G-CSF receptor binding experiment was conducted as follows. $Na^{125}I$ (Dupon NEN) and enzymo-bead reagent (Bio-Rad) were used. Recombinant mutant G-CSF (KW-2228), kindly provided by Kyowa Hakko Kogyo, Japan, was employed as the ligand. KW-2228 was radioiodinated with 37 MBq of $Na^{125}I$ by using the solid-phase glucose oxidase–lactoperoxidase method as described by Piao et al (1990). The specific activity of radioiodinated KW-2228 was $6 \times 10^6$ c.p.m. $\mu g^{-1}$ protein. The cultured cells were incubated for 24 h at 4°C in 24-well tissue culture plates in 0.5 ml of isotonic phosphate-buffered saline (PBS) containing 0.2% bovine serum albumin and $^{125}I$-labelled KW-2228 with or without KW-2228. Following incubation for 24 h, the medium was aspirated, and the cells were washed with cold PBS. The cells were then solubilized in 0.25 ml of 2M sodium hydroxide, and the radioactivities were measured. Non-specific binding was measured in the presence of G-CSF at 1000 ng per 0.5 ml. The Scatchard plot of the specific binding of $^{125}I$-labelled KW-2228 to the cells was then estimated to obtain the affinity and receptor numbers.

The stimulation of cultured cancer cells by exogenous G-CSF administration under serum-free conditions was studied. To maintain and subculture these cell lines under serum-free condition, we needed some preparation and modification of cell cultures. First of all, these cells were subcultured with gradient degrees of lower serum supplementations and then were finally subcultured in a serum-free medium with the supplementation of transferrin and insulin (insulin–transferrin–sodium selenite media supplement, Sigma). After completing the preparation, these cells can be maintained and subcultured under serum-free conditions. The estimated viability of these cells under serum-free conditions by the trypan-blue dye exclusion method was more than a 83% viability (maximum 97.9%), which was not different from the serum supplement conditions. The proliferating activity of the cultured cancer cells was measured using a flow cytometric bromodeoxyuridine incorporation technique as previously described elsewhere (Tachibana et al, 1995). Briefly, the $3 \times 10^4$ cells were incubated in 1 ml of RPMI 1640 medium (Gibco BRL) without serum supplementation in 12-well culture dishes (Corning, New York; well diameter 22 mm) at 37°C in a humidified atmosphere of 5% carbon dioxide with 95% air. Recombinant mutant human G-CSF was added at a final concentration of 0.5 μg $ml^{-1}$ every 24 h for a total of three times. Twenty-four hours after the final G-CSF treatment (72 h after cell culture), bromodeoxyuridine was added to each culture well at a final concentration of 5 μg $ml^{-1}$, and then the incubation was continued for a further 1 h. The cells were harvested with 0.25% trypsin with 1 mM ethylenediaminetetraacetic acid (EDTA) and were then washed twice. The cells were subsequently stained by fluorescein isothiocyanate (FITC)-labelled anti-bromodeoxyuridine antibody and then post-stained by 0.5% propidium iodide. The double-stained cells were analysed by an Epics Elite flow cytometer (Coulter, USA), and the labelling index (LI), i.e. the number of cells stained by bromodeoxyuridine divided by the total estimated cell count, was calculated.

Next, the same experiments were carried out using the [$^3$H]thymidine incorporation method. The serum-free subclone cells ($1 \times 10^4$) were incubated in 0.1 ml of the culture medium without fetal calf serum (FCS) in a 96-well microtitre tray (Nunc, Denmark). Recombinant G-CSF was added every 24 h at a final concentration of 0.5 μg $ml^{-1}$ for a total of three times to the cell cultures. Twenty-four hours after the final G-CSF treatment, DNA synthesis in the cultures was determined by the addition of [methyl-$^3$H]thymidine (Amersham, UK) (0.6 μCi per well; 1 Ci=37 MBq) during a 4-h pulse. The cells were then harvested onto glass fibre filters and counted by liquid scintillation counter (LS 9800, Beckman Instruments, USA).

All data are expressed as the mean ± s.d. The difference was determined by Student's *t*-test (two-tailed) and $P < 0.05$ was regarded as statistically significant.

### Tumour tissues specimens

Twenty-six tumour tissue specimens from patients with histologically confirmed transitional cell carcinoma of the bladder were used in this study. Both normal tissue and tumour samples were obtained by transurethral cold-cup biopsy technique from each patient. Frozen sections cut at 5 μm were fixed with Streck Tissue Fixative (STF, Streck Laboratories, Omaha, NE, USA) for 5 min before processing for the in situ detection of G-CSF receptor mRNA.

### In situ detection of G-CSF receptor mRNA signals by RT-PCR

The in situ RT-PCR detection of G-CSF receptor mRNA on tumour samples was performed according to the modified method as described by Nuovo et al (1995).

Briefly, frozen sections following STF fixation were digested with proteinase K at 30 μg $ml^{-1}$ in 20 mM Tris-HCl, pH 7.4, for 5 min. The tissue specimens were then treated overnight with an RNAase-free DNAase solution made according to the manufacturer's recommendations at 37°C. The tissue specimens were incubated directly on a glass slide at 42°C for 30 min with 10 μl of a solution containing the downstream primer (1 μM) and reverse transcriptase (5 units; RT-PCR Kit, Perkin Elmer, Norwalk, CT, USA). Each case was analysed for the expression of G-CSF receptor transcripts. The sequences of the primer used for the detection of the corresponding cDNA was previously described in the section covering the RT-PCR method.

The solution for the amplification of the cDNA contained 4.5 mM magnesium chloride, 200 μM each of dATP, dCTP, dGTP

© Cancer Research Campaign 1997

and dTTP (for digoxigenin), 1 μM of each primer, 100 μg $ml^{-1}$ of BSA and 5 units *Taq* polymerase (Perkin Elmer) per 40 μl of amplifying solution. Digoxigenin dUTP was used as the reporter molecule. The concentration of digoxigenin in the amplifying solution was 10 μM. The digoxigenin-labelled PCR product was detected after incubation with an alkaline phosphatase–antidigoxigenin conjugate (1:200 dilution in 0.1 M Tris-HCl, pH 7.4) and 0.1 M sodium chloride for overnight at 37°C. Staining development was then performed using chromagen nitroblue tetrazolium and 5-bromo-4-chloro-3-indol-phosphate for 5 min. The PCR reaction was performed with an initial denaturing step of 94°C for 3 min followed by 25 cycles of annealing extension at the same temperatures and times as previously described in the section concerning the RT-PCR for the G-CSF receptor.

One essential aspect of this protocol is that the negative and positive controls can be performed on the same glass slide along with the experimental analysis. The positive control for in situ PCR eliminates the DNAase digestive reaction. An intense nuclear signal is generated from the target-specific amplification, DNA repair and mispriming. This control demonstrates that the PCR reaction and the subsequent detection steps all worked successfully. A prerequisite for the successful amplification of any given cDNA is not a true positive control for the G-CSF receptor mRNA but rather an intense signal with the in situ RT-PCR-positive control. The negative control constitutes in situ RT-PCR in which the tissue is treated with DNAase and the RT step is eliminated. The absence of a signal thus demonstrates that amplification of genomic DNA does not occur. The results for G-CSF receptor signal expressions were read blindly by J-IH (a pathologist) and MT and AM (urologists). If any obviously positive signal expressions were observed on the tumour cells, the results were designated as positive. On the other hand, if no definitively positive signal expressions were seen on the tumour cells, then the results were defined as negative. When all three examiners agreed with the positive and/or negative results, the final results were thus defined as positive (+) and/or negative (–). However, when the results differed among the examiners, the final results were designated as inconclusive (±).

### RESULTS

G-CSF receptor m-RNA expressions of the cultured cancer cell lines were studied using the RT-PCR method. The RT-PCR product exhibited a specific G-CSF receptor transcription signal of 340 bp in the samples from the cultured cells of KU-1, NBT-2 and KK (Figure 1).

Binding studies using radiolabelled recombinant G-CSF demonstrated the presence of high-affinity G-CSF binding receptors on the cultured cancer cell lines of KU-1, NBT-2 and KK. Non-specific binding, which ranged between 5% and 22%, was subtracted from the total binding to determine the specific binding. The Scatchard plot of the specific binding of $^{125}$I-labelled KW-2228 to the cell lines indicated that the cells harbour a single type of G-CSF receptor (Figure 2). The $B_{max}$ calculated from the Scatchard plots were 3140, 4010 and 458 molecules per cell for KU-1, NBT-2 and KK respectively. The equilibrium dissociation constants ($K_d$) of KU-1, NBT-2 and KK were 490 pM, 340 pM and 103 pM respectively.

The results of exogenous G-CSF stimulation obtained by both flow cytometric BrdUrd labelling and [$^3$H]thymidine incorporation methods are listed in Table 2.

**Figure 1** Detection of G-CSF receptor mRNA expressions of the cultured cancer cell lines by RT-PCR method. The RT-PCR product exhibited a specific G-CSF receptor transcription signal of 340 bp in samples from the cultured cells of KU-1 (**A**), NBT-2 (**B**) and KK (**C**). N, negative control by minus reverse transcriptase PCR; M, markers; P, positive control; A, KU-1; B, NBT-2; C, KK. Size markers from top, 2,072, 1,500, then every 100 bp

**Figure 2** Scatchard plot analyses of the specific binding of $^{125}$I-labelled recombinant G-CSF to the cultured cell lines. The $B_{max}$ values, obtained from the intercept of the slope with the abscissa on the Scatchard plots, were 490 pM, 340 pM and 103 pM for KU-1, NBT-2 and KK respectively. B/F, bound–free ratio

The BrdUrd labelling of KU-1, NBT-2 and KK at 72 h after initial incubation with 0.5 μg $ml^{-1}$ G-CSF were 31.2 ± 2.1% in KU-1, 22.5 ± 3.8% in NBT-2, 18.8 ± 2.2% in KK, 11.2 ± 1.2% in KU-7 and 15.8 ± 1.9% in T-24. The BrdUrd labelling of KU-1, NBT-2 and KK with G-CSF were significantly higher than those of controls (without G-CSF administration), i.e. 21.3 ± 2.4% in KU-1, 16.5 ± 3.1% in NBT-2 and 11.4 ± 1.8% in KK ($P < 0.01$). However, KU-7 and T-24 did not demonstrate any increased BrdUrd labelling when compared with the controls i.e. 10.5 ± 1.2% in KU-7 and 16.7 ± 2.5% in T-24.

© Cancer Research Campaign 1997

Table 2 The results of exogenous G-CSF stimulation obtained by both flow cytometric bromodeoxyuridine (BrdUrd) labelling and [$^3$H]thymidine incorporation methods.

| | BrdUrd labelling (Z) | | [$^3$H]Thymidine incorporation (c.p.m. per well) | |
|---|---|---|---|---|
| | Control | G-CSF stimulation | Control | G-CSF stimulation |
| KU-1 | 21.3 ± 2.4 | 31.2 ± 2.1** | 6168.0 ± 441.8 | 6900.3 ± 308.4* |
| NBT-2 | 16.5 ± 3.1 | 22.5 ± 3.8* | 5004.4 ± 218.2 | 5483.8 ± 302.7* |
| KK | 11.4 ± 1.8 | 18.8 ± 2.2** | 4475.2 ± 213.3 | 5217.3 ± 480.9** |
| KU-7 | 10.5 ± 1.2 | 11.2 ± 1.2 | 4057.8 ± 216.2 | 4089.5 ± 306.2 |
| T-24 | 16.7 ± 2.5 | 15.8 ± 1.9 | 5353.2 ± 249.6 | 5308.0 ± 458.3 |

The BrdUrd labelling of KU-1, NBT-2 and KK at 72 h after initial incubation with 0.5 μg ml$^{-1}$ G-CSF were 31.2 ± 2.1% in KU-1, 22.5 ± 3.8% in NBT-2, 18.8 ± 2.2% in KK, 11.2 ± 1.2% in KU-7 and 15.8 ± 1.9% in T-24. The BrdUrd labelling of KU-1, NBT-2 and KK with G-CSF were significantly higher than those of controls (without G-CSF administration) (21.3 ± 2.4% in KU-1, 16.5 ± 3.1% in NBT-2 and 11.4 ± 1.8% in KK) ($P < 0.05$). However, KU-7 and T-24 did not demonstrate any increased BrdUrd labelling when compared with the controls (10.5 ± 1.2% in KU-7 and 16.7 ± 2.5% in T-24). In addition, exactly the same results were also obtained using the [$^3$H]thymidine incorporation method. KU-1, NBT-2 and KK cells exhibited higher [$^3$H]thymidine incorporations than those without G-CSF stimulation. The differences were also statistically significant ($P$<0.05 for KU-1 and NBT-2, $P$<0.01 for KK). BrdUrd, bromodeoxyuridine; G-CSF, granulocyte colony-stimulating factor; c.p.m., count per minute; *$P$ <0.05; **$P$ <0.01.

In addition, exactly the same results were also obtained using the [$^3$H]thymidine incorporation method. KU-1, NBT-2 and KK cells exhibited higher [$^3$H]thymidine incorporations than those without G-CSF stimulation. The differences were also statistically significant ($P$<0.05 for KU-1 and NBT-2, $P$<0.01 for KK).

Twenty-six cases of human bladder cancer were included in this study and their salient pathological data and the presence or absence of expression of G-CSF receptor mRNA signals obtained by in situ RT-PCR are also provided in Table 3. With in situ RT-PCR, the tumour cells of 6 of the 26 bladder tumour specimens (23.1%) had a positive G-CSF receptor mRNA signal. On the other hand, 17 tumour specimens demonstrated negative signals in three specimens, thus resulting in inconclusive findings (±).

The G-CSF receptor expressions on five different cultured cell lines were also determined using the in situ RT-PCR method. Three out of the five cultured cell lines (KU-1, NBT and KK) also exhibited positive results for the G-CSF receptor mRNA signals, the same as for the conventional RT-PCR method. Figure 3A demonstrates the detection of G-CSF receptor expression of cultured cancer cells (KK) by the in situ RT-PCR method. The signal in the nuclei of many of the cancer cells for G-CSF receptor was also noted. The signal was lost in the material when the reverse transcriptase reaction step was omitted (Figure 3B).

Figure 4 presents an in situ RT-PCR specimen of Case 3 and the histological findings, classifying it as a grade 3, muscle-invasive transitional cell carcinoma of the bladder (Figure 4A). The papillary part of the tumour cells exhibited a strongly stained, relatively homogenous pattern based on the in situ RT-PCR method (Figure 4B). In addition, the submucosal tissue clearly demonstrated negative staining.

It was also interesting to note that when the expression of G-CSF receptor mRNA on tumour tissue was compared with the histological tumour grades and stages, the staining results did not correlate with the tumour grade or stage.

Table 3 Summary of granulocyte colony-stimulating factor receptor (G-CSFR) expression of human bladder cancer tissues determined by in situ reverse transcriptase–polymerase chain reaction.

| Case | Grade | Stage | G-CSFR expression |
|---|---|---|---|
| 1 | 3 | pT3b | + |
| 2 | 2 | pT1b | + |
| 3 | 3 | pT3b | + |
| 4 | 2 | pT1b | + |
| 5 | 2 | pT1a | + |
| 6 | 2 | pT1a | + |
| 7 | 3 | pT4 | – |
| 8 | 2 | pT1a | – |
| 9 | 3 | pT3b | – |
| 10 | 3 | pT4 | – |
| 11 | 2 | pTa | – |
| 12 | 3 | pT3b | – |
| 13 | 3 | pT4 | – |
| 14 | 2 | pT1a | – |
| 15 | 2 | pT1a | – |
| 16 | 1 | pT1a | – |
| 17 | 3 | pT4 | – |
| 18 | 2 | pT1b | – |
| 19 | 2 | pT1a | – |
| 20 | 1 | pT1a | – |
| 21 | 2 | pT1b | – |
| 22 | 2 | pT1a | – |
| 23 | 3 | pT2 | – |
| 24 | 2 | pT1b | ± |
| 25 | 2 | pT1a | ± |
| 26 | 3 | pT3b | ± |

With in situ RT-PCR, the tumour cells of 6 of the 26 bladder tumour specimens (23.1%) had a positive G-CSF receptor mRNA signal. On the other hand, 17 tumour specimens demonstrated negative signals in three specimens, thus resulting in inconclusive findings (±).

### DISCUSSION

Although the incorporation of systemic chemotherapy in the treatment of patients with invasive bladder cancer is becoming an extremely important treatment modality, the majority of morbidities are from bone marrow suppression, which may be the main cause of the dose-limiting factor, resulting in a systemic relapse (Scher, 1992). The availability of haematopoietic growth factors has reduced the toxicities of the regimens currently in use. In the earliest trial of granulocyte colony-stimulating factor and M-VAC chemotherapy for the treatment of urothelial cancers, such as the coadministration of haematopoietic growth factors associated with combination chemotherapy, the treatment cycles with and without the growth factor were compared in each individual. When the

 © Cancer Research Campaign 1997

**Figure 3** Detection of G-CSF receptor expression of cultured cancer cells (KK) by the in situ RT-PCR method. Note the signal in the nuclei of many of the cells for G-CSF receptor (**A**, × 200). This signal was lost in the material if the reverse transcriptase step was omitted (**B**, × 200)

patients were able to receive full doses of the drugs scheduled at interval days 14 and 21 (100% versus 29%), fewer days (3 vs 32) of neutropenia were observed, and the incidence of mucositis was also reduced with the coadministration of the G-CSF (Gabrilove et al, 1988*b*).

Subsequent studies have further confirmed the efficacy of G-CSF in conjunction with systemic chemotherapy for advanced bladder cancer patients (Aso et al, 1992; Miyanaga et al, 1994). Therefore, reducing the toxicities of the chemotherapy ensures that treatment recommendations will allow patients the maximal opportunity for both a cure and the maintenance of their organ functions, while, at the same time, minimizing the toxicities in patients for whom systemic approaches are unwarranted. Interestingly, it has been reported recently that G-CSF may enhance tumour sensitivity to methotrexate in vitro (Ohigashi, 1990) and increase the sensitivity of implanted urothelial tumours to chemotherapy in nude mice (Akaza et al, 1992). Furthermore, a significantly additive inhibitory effect on the in vitro cell growth of the human urothelial tumour cell line (EJ28) under the combined administration of G-CSF and methotrexate has also been reported (Block et al, 1993). These findings thus suggest an expanded role for these agents. Furthermore, the high-dose chemotherapy administered as part of bone marrow transplants produces a prolonged aplastic period, entailing a high risk of life-threatening status. In this regard, recombinant G-CSF has been studied in patients undergoing autologous and allogeneic transplants. In both autologous and allogeneic transplant settings, G-CSF accelerated myeloid recovery compared with the historical control (Masaoka et al, 1989; Taylor et al, 1989).

More recently, it has been shown that patients who are in need of a bone marrow transplant but who cannot be harvested because of bone marrow tumour involvement or pelvic radiation-induced marrow injury may now undergo such transplants using stem cells collected from the peripheral blood. Indeed, peripheral stem cell transplants have been successfully performed in patients with Hodgkin's disease, non-Hodgkin's lymphoma, myeloma and other solid cancers, such as breast, ovarian and testis cancer and in children with neuroblastomas (Kessinger et al, 1986, 1989; Juttner et al, 1988; Fremand et al, 1989; Lasky et al, 1989). Therefore, the application of G-CSF in combination with chemotherapy has become widely accepted as a treatment modality.

It should be pointed out, on the other hand, that G-CSF receptor expressions have been found on the surface of several non-haematopoietic cell types, including human carcinoma cells (Bussolino et al, 1989; Uzumaki et al, 1989; Avaros et al, 1990). In fact, we previously reported that human transitional carcinoma cells expressing G-CSF receptor generated an acceleration of tumour cell proliferation (Ohigashi et al, 1992; Tachibana et al, 1995).

© Cancer Research Campaign 1997

Figure 4 Detection of G-CSF receptor expression in human cancer tissues by the in situ RT-PCR method. Haematoxylin and eosin staining of the tissue (**A**, × 200). Note the signal in the nuclei of many of the cancer cells for G-CSF receptor (**B**, × 200). This signal was lost in the serial section if the reverse transcriptase step was omitted (**C**, × 200)

The above observations have naturally led to the tempting speculation that G-CSF systemic administration may provide growth promotion for transitional carcinoma cells. Thus, the frequency of G-CSF receptor expression on transitional cell carcinoma cells has become an extremely significant factor with respect to the application of G-CSF in a clinical setting.

As was observed in this study, G-CSF receptor expressions were frequently observed on human transitional carcinoma cells of the bladder. However, the physiological significance of G-CSF receptors on the surface of non-haematopoietic cells remains unclear. Controversy exists concerning whether or not these G-CSF receptors are similar to the receptors expressed on cells of neutrophilic lineage, binding G-CSF with high affinity, and whether they are present on the surface of these non-haematopoietic cell types in sparse numbers (Bussolino et al, 1989; Uzumaki et al, 1989; Avaros et al, 1990). Moreover, at present, five different human G-CSF receptor isoforms or classes arising from alternative RNA splicing have been isolated, which are all identical in the extracellular domain but differ in their downstream sequences (Avalos et al, 1996). The physiological roles of the various human G-CSF receptor isoforms and the regulation of their expression remain unclear, although the class II soluble receptor may function to negatively modulate responses to G-CSF by serving as a non-signalling

 © Cancer Research Campaign 1997

sink that directly competes with the membrane-bound receptor forms in a dose-dependent fashion for ligand binding, as has been reported for the soluble receptor for epidermal growth factor (Basu, 1996). In contrast, the class II receptor could positively modulate the responses to G-CSF by serving as a binding protein that protects G-CSF from degradation or clearance in the extracellular space, similar to the growth hormone binding protein that has been shown to prolong the overall half-life of growth hormones (Veldhuis et al, 1993). Therefore, the expression of G-CSF receptor does not necessarily positively modulate the responses to G-CSF.

In line with these findings, our previous observation demonstrated that the G-CSF-expressing bladder cancer cells exhibited high-affinity binding to G-CSF and promoted the growth stimulation of cultured bladder cancer cells.

In addition, numerous studies have demonstrated that some human bladder cancers may produce G-CSF frequently, presenting leukaemoid reactions (Mizutani et al, 1995). Furthermore, G-CSF production in vitro was demonstrated in another bladder cancer cell line (5637) that is used as a source of G-CSF (Bailly et al, 1995).

Taking all these things into consideration, G-CSF and its receptor expression may therefore act in tumour cell paracrine and/or autocrine loop mechanisms.

This observation therefore suggests that G-CSF may stimulate the clonal growth of human bladder cancer cells by binding to its specific receptors if these cells feature G-CSF receptors on the surface. It should be pointed out, however, that this finding does not diminish the efficacy of G-CSF administration in conjunction with systemic chemotherapy, as chemotherapy does work extremely well for such cancers as testis cancer and some haematopoietic cancers.

At this time, we can only recommend that all bladder cancers should be tested for the expression of G-CSF receptor before using G-CSF, because there is the possibility of growth promotion by G-CSF if they have G-CSF receptor on their cell surfaces.

The in situ determination of the G-CSF receptor expression on the surface of these cells may be difficult because the previous technique using standard in situ hybridization is a far less sensitive method than PCR techniques. It is obvious that greater sensitivity can be obtained by employing the in situ RT-PCR technique than by using standard RNA with in situ hybridization subsequent to the 10- to 100-fold increase in the target copy number following amplification (Mignatti et al, 1986; Nuovo et al, 1993). Therefore, the significant advantages of this technique are its capabilities of detecting very minute mRNA expression on the cells and in clarifying the exact source of the particular cells. Thus, this in situ RT-PCR method is considered to be a useful technique for studying the relationship between tumour behaviour and cytokine and/or cytokine receptor expression.

### ACKNOWLEDGEMENT

This work was supported in part by Grants-in-Aid nos. 07407046 and 10146673 for Scientific Research from the Ministry of Education, Science and Culture, Japan.

### REFERENCES

Akaza H, Fukushima H, Koiso K and Aso Y (1992) Enhancement of chemotherapeutic effects by recombinant human granulocyte colony-stimulating factor on implanted mouse bladder cancer cells (MBT-2). *Cancer* **69**: 997–1002

Aso Y and Akaza H (1992) Urological rhG-CSF study group: effect of recombinant human granulocyte colony-stimulating factor in patients receiving chemotherapy for urological cancer. *J Urol* **147**: 1060–1064

Avalos BR, Gasson JC, Hedvat C, Quan SG, Baldwin GC, Weisbart RH, Williams RE, Golde DW and DiPersio JF (1990) Human granulocyte colony-stimulating factor: biologic activities and receptor characterization on hematopoietic cells and small cell lung cancer cell lines. *Blood* **75**: 851–857

Avalos BR (1996) Molecular analysis of the granulocyte colony-stimulating factor receptor. *Blood* **88**: 761–777

Bailly J-D, Pourquier P, Jaffrezou J-P, Duchayne E, Cassar G, Bordier C and Laurent G (1995) Effect of 5637-conditioned medium and recombinant cytokines on P-glycoprotein expression in a human GM-CSF-dependent leukemic myeloid cell line. *Leukemia* **9**: 1718–1725

Basu A, Raghunath M, Bishayee S and Das M (1989) Inhibition of tyrosine kinase activity of the epidermal growth factor (EGF) receptor by a truncated receptor from that binds to EGF: role for interreceptor interaction in kinase regulation. *Mol Cell Biol* **9**: 671–677

Begley CG, Metcalf D and Nicola NA (1987) Primary human myeloid leukemia cells: comparative responsiveness to proliferative stimulation by GM-CSF or G-CSF and membrane expression of CSF receptors. *Leukemia* **1**: 1–8

Block T, Treiber U, Geffken B, Vogel M, Hanauske A-R, Schmid F, Hartung R and Busch R (1993) Inhibitory effects on in vitro cell growth of human urothelial tumor cell lines under the combined administration of hematopoietic growth factors and clinically relevant antineoplastic agents. *Urol Res* **21**: 217–221

Bussolino F, Wang JM, Defilippi P, Turrini F, Sanavio F, Edgell CJ, Aglietta M, Arese P and Matovani A (1989) Granulocyte- and granulocyte–macrophage-colony stimulating factors induce human endothelial cells to migrate and proliferate. *Nature* **337**: 471–473

Chomozynski P and Sacchi N (1987) Single-step method of RNA isolation by acid guanidium thiocyanate–phenol–chloroform extraction. *Anal Chem* **162**: 156–159

Crawford J, Ozer H, Stoller R, Johnson D, Lyman G, Tabbara I, Kris M, Grous J, Picozzi V, Rausch G, Smith R, Gradishar W, Yahanda A, Vincent M, Stewart M and Glaspy J (1991) Reduction by granulocyte colony-stimulating factor of fever and neutropenia induced by chemotherapy in patients with small-cell lung cancer. *N Engl J Med* **315**: 164–170

Demetri GD and Griffin JD (1991) Granulocyte colony-stimulating factor and its receptor. *Blood* **78**: 2791–2808

Fremand J-P, Levy Y, Gerota J, Benbunan M, Casset JM, Castaigne S, Seligmann M and Brouet J-C (1989) Treatment of aggressive multiple myeloma by high-dose chemotherapy and total body irradiation followed by blood stem cells autologous graft. *Blood* **73**: 20–23

Gabrilove J (1991) Hematopoietic growth factors in the supportive care and treatment of patients with hematologic malignancies. In *Neoplastic Diseases of the Blood*. Wiernick PH, Canellos GP, Kyle RA and Schiffer CA. (eds), pp. 949–965. Churchill Livingstone: New York, NY

Gabrilove JL, Jakubowski A, Scher H, Sternberg C, Wong G, Grous J, Yagoda A, Fain K, Moore MAS, Clarkson B, Oettgen HF, Alton K, Welte K and Souza L (1988*a*) Effect of granulocyte colony-stimulating factor on neutropenia and associated morbidity due to chemotherapy for transitional-cell carcinoma of the uroepithelium. *N Engl J Med* **318**: 1414–1422

Gabrilove JL, Jakubowski A, Fain K, Grous J, Scher H, Sternberg C, Yagoda A, Clarkson B, Bonilla MA, Oettgen HF, Alton K, Boone T, Altrock B, Welte K and Souza L (1988*b*) Phase I study of granulocyte colony-stimulating factor in patients with transitional cell carcinoma of the urothelium. *J Clin Invest* **82**: 1454–1461

Juttner CA, To LB, Ho JQK, Bardy PG, Dyson PG, Haylock DN and Kimber RJ (1988) Early lympho-hematopoietic recovery after autografting using peripheral blood stem cells in acute non-lymphoblastic leukemia. *Trans Proc* **20**: 40–43

Kessinger A, Armitage JO, Landmark JD and Weisenburger DD (1986) Reconstitution of human hematopoietic function with autologous, cryopreserved circulating stem cells. *Exp Hematol* **14**: 192–196

Kessinger A, Armitage JO, Smith DM, Landmark JD, Bierman PJ and Weisenburger DD (1989) High-dose therapy and autologous peripheral blood stem cell transplantation for patients with lymphoma. *Blood* **74**: 1260–1266

Lasky LC, Bostrom B, Smith J, Moss, TJ and Ramsay NKC (1989) Clinical collection and use of peripheral blood stem cells in pediatric patients. *Transplantation* **47**: 613–616

Masaoka T, Takaku F, Kato S, Moriyamam Y, Kodera Y, Kanamura A, Shimosato A, Shibata H and Nakamura H (1989) Recombinant human granulocyte colony-stimulating factor in allogenic bone marrow transplantation. *Exp Hematol* **17**: 1047–1050

Mignatti P, Robbins E and Rifkin DB (1986) Tumor invasion through the human amniotic membrane: requirement for a protease cascade. *Cell* **47**: 487–498

© Cancer Research Campaign 1997

Miyanaga N, Akaza H, Shimazui T, Ohtani M and Koiso K (1994) The effect of dose intensity on M-VAC therapy for advanced urothelial cancer. *Cancer Chemother Pharmacol* **35**: (suppl.) S5–S8

Mizutani Y, Okada Y, Terachi T, Kakei Y and Yoshida O (1995) Serum granulocyte colony-stimulating factor levels in patients with urinary bladder tumour and various urological malignancies. *Br J Urol* **76**: 580–586

Morstyn G, Campbell L, Souza LM, Alton NK, Keech J, Green M, Sheridan W, Metcalf D and Fox R (1988) Effect of granulocyte colony stimulating factor on neutropenia induced by cytotoxic chemotherapy. *Lancet* **1**: 667–672

Nicola NA, and Metcalf D (1985) Binding of $^{125}$I-labeled granulocyte colony-stimulating factor to normal murine hematopoietic cells. *J Cell Physiol* **124**: 313–321

Nuovo GJ, Lidonocci K, MacConnell P and Lane B (1993) Intracellular localization of polymerase chain reaction-amplified hepatitis C cDNA. *Am J Surg Pathol* **17**: 683–690

Nuovo GJ, MacConnell PB, Simsir A, Valea F and French DL (1995) Correlation of the in situ detection of polymerase chain reaction-amplified metalloproteinase complementary DNAs and their inhibitors with prognosis in cervical carcinoma. *Cancer Res* **55**: 267–275

Ohigashi T (1990) Granulocyte-colony stimulating factor enhances the cytotoxic effects of methotrexate to bladder cancer cells in vitro. *Keio J Med* **39**: 254–260

Ohigashi T, Tachibana M, Tazaki H and Nakamura K (1992) Bladder cancer cells express functional receptors for granulocyte-colony stimulating factor. *J Urol* **147**: 283–286

Ohno R, Tomonaga M, Kobayashi T, Kanamaru A, Shirakawa S, Masaoka T, Omine M, Oh H, Nomura T, Sakai Y, Hirano M, Yokomaru S, Nakayama S, Yoshida Y, Miura AB, Morishita Y, Dohy H, Niho Y, Hamajima N and Takaku F (1990) Effect of granulocyte colony-stimulating factor after intensive induction therapy in relapsed or refractory acute leukemia. *N Engl J Med* **323**: 871–877

Park LS, Waldron PE, Friend D, Sassenfeld HM, Price V, Anderson D, Cosman D, Andrews RG, Bernstein ID and Urdal DL (1989) Interleukin-3, GM-CSF and G-CSF receptor expression on cell lines and primary leukemia cells. *Blood* **74**: 56–65

Piao Y-F and Okabe T (1990) Receptor binding of human granulocyte colony-stimulating factor to the blast cells of myeloid leukemia. *Cancer Res* **50**: 1671–1674

Scher HI (1992) Systemic chemotherapy in regionally advanced bladder cancer: theoretical considerations and results. *Urol Clin North Am* **19**: 747–759

Tachibana M (1982) Studies on cellular adhesiveness in five different culture cell lines derived from carcinoma of the urinary bladder. Keio J Med **31**: 127–148

Tachibana M, Miyakawa A, Tazaki H, Nakamura K, Kubo A, Hata J, Nishi T and Amano Y (1995) Autocrine growth of transitional cell carcinoma of the bladder induced by granulocyte-colony stimulating factor. *Cancer Res* **55**: 3438–3443

Taylor KM, Jagannath S, Spitzer G, Spinolo JA, Tucker SL, Fogel B, Cabanillas FF, Hagemeiste FB and Souza LM (1989) Recombinant human granulocyte colony-stimulating factor hastens granulocyte recovery after high-dose chemotherapy and autologous bone marrow transplantation in Hodgkin's disease. *J Clin Oncol* **7**: 1791–1799

Uzumaki H, Okabe T, Sasaki N, Hagiwara K, Takaku F, Tobita M, Yasukawa K, Ito S and Umezawa Y (1989) Identification and characterization of receptors for granulocyte colony-stimulating factor on human placenta and trophoblastic cells. Proc Natl Acad Sci USA **86**: 9323–9326

Veldhuis JD, Johnson ML, Faunt LM, Mercado M and Baumann G (1993) Influence of the high-affinity growth hormone (GH)-binding protein on plasma profiles free and bound GH and on the apparent half-life of GH. *J Clin Invest* **91**: 629–641

© Cancer Research Campaign 1997